# Veillonellae: Beyond Bridging Species in Oral Biofilm Ecology

**DOI:** 10.3389/froh.2021.774115

**Published:** 2021-10-29

**Authors:** Peng Zhou, Daniel Manoil, Georgios N. Belibasakis, Georgios A. Kotsakis

**Affiliations:** ^1^Translational Periodontal Research Lab, Department of Periodontics, School of Dentistry, UT Health San Antonio, San Antonio, TX, United States; ^2^Division of Oral Diseases, Department of Dental Medicine, Karolinska Institute, Huddinge, Sweden

**Keywords:** veillonellae, accessory pathogens, bridging species, oral biofilm, dental caries, periodontitis, peri-implantitis

## Abstract

The genus *Veillonella* comprises 16 characterized species, among which eight are commonly found in the human oral cavity. The high abundance of *Veillonella* species in the microbiome of both supra- and sub-gingival biofilms, and their interdependent relationship with a multitude of other bacterial species, suggest veillonellae to play an important role in oral biofilm ecology. Development of oral biofilms relies on an incremental coaggregation process between early, bridging and later bacterial colonizers, ultimately forming multispecies communities. As early colonizer and bridging species, veillonellae are critical in guiding the development of multispecies communities in the human oral microenvironment. Their ability to establish mutualistic relationships with other members of the oral microbiome has emerged as a crucial factor that may contribute to health equilibrium. Here, we review the general characteristics, taxonomy, physiology, genomic and genetics of veillonellae, as well as their bridging role in the development of oral biofilms. We further discuss the role of *Veillonella* spp. as potential “accessory pathogens” in the human oral cavity, capable of supporting colonization by other, more pathogenic species. The relationship between *Veillonella* spp. and dental caries, periodontitis, and peri-implantitis is also recapitulated in this review. We finally highlight areas of future research required to better understand the intergeneric signaling employed by veillonellae during their bridging activities and interspecies mutualism. With the recent discoveries of large species and strain-specific variation within the genus in biological and virulence characteristics, the study of *Veillonella* as an example of highly adaptive microorganisms that indirectly participates in dysbiosis holds great promise for broadening our understanding of polymicrobial disease pathogenesis.

## Introduction

The human oral cavity is home to one of the richest microbiotas of the human body, one primarily dominated by the domain Bacteria. Thus far, no <16 different bacterial Phyla comprising 106 Families, 231 Genera and 687 species-level taxa were cataloged in the Human Oral Microbiome Database (HOMD) [1]. This microbiota is almost exclusively present as biofilm-growing polymicrobial communities [2]. Whereas, these biofilms may evolve in homeostasis with the human host, they are also the cause of two of the most prevalent diseases of mankind: dental caries and periodontitis [3, 4].

Human studies conducted in the past half century have strived to identify specific pathogens implicated in the pathogenesis of these diseases. As such, Mutans streptococci (mainly *Streptococcus mutans*) were depicted as the main causal agents of dental caries [5], and species of the so-called “red complex”, i.e., *Porphyromonas gingivalis, Treponema denticola*, and *Tannerella forsythia* were described as major etiological drivers of periodontitis [6]. Although these organisms have successfully served as models to enhance our understanding of the bacterial processes involved in oral diseases, the more recent advent of high-throughput sequencing technologies has greatly expanded the catalog of taxa identified within oral communities and further broadened our understanding of their ecological function and contribution to these diseases [7]. Compelling evidence now demonstrates that not only are these diseases polymicrobial in nature but also that they require polymicrobial synergism both to be initiated and progress [8]. Viewing oral diseases through the prism of single causative taxa was therefore rapidly deemed insufficient and gave way to the study of microbiota symbiosis and dysbiosis with host tissues. A cardinal feature of polymicrobial diseases, such as periodontitis, is that the compositional and functional activity of the local microbiota is highly responsive to environmental dynamics. Consequently, oral taxa that are widely regarded as being commensal and part of the healthy state microbiota may, in fact, demonstrate critical roles in compositional shifts of the normal microbiota from symbiosis to dysbiosis when the microenvironmental changes provide such opportunities.

Understanding the early events that lead to dysbiosis is a major focus area of current research efforts toward using holistic means for disease prevention, and precisely, representatives of the genus *Veillonella* are purported to play a major role in the ecology of oral biofilms [9–11]. Despite representing among the most abundant genus in the oral cavity, after *Streptococcus* and *Prevotella* [12], *Veillonella* spp. remain poorly studied. In the words of Kolenbrander, veillonellae have emerged as “*a critical genus that guides the development of multispecies communities*” [13]. In the current review, we recapitulate information on the taxonomy, physiology, genomic and genetics of the genus *Veillonella*, and further provide nascent evidence that suggest the possibility that *Veillonella* species may act as “accessory pathogens”, i.e., have the capabilities to promote the growth of more pathogenic species within oral biofilms, finally epitomize the relationship between these bacteria and biofilm-induced oral diseases—dental caries and periodontitis.

## Microbial Ecology in the Oral Cavity: The Pivotal Position of *Veillonella* Species

The oral cavity represents a unique ecosystem spatially and ecologically diverse. This ecosystem offers epithelial surfaces alternating both keratinized and non-keratinized profiles, as well as coarser and cryptic mucosal surfaces such as on the tongue or tonsils. In addition to shedding epithelia, dental tissues expose non-shedding hard surfaces that display both accessible supragingival areas and protected subgingival surfaces. This interface between dental and gingival tissues creates a crevice, the gingival sulcus, which is physiologically bathed in a serum-derived, nutrient-rich transudate [14, 15]. Such spatial diversity creates local gradients of temperature, redox potential, pH and nutrients sinks, all of which create ecologically different niches that differently support the growth of microbial communities [16]. On top of that, an additional ecological pressure stems from the constant salivary flow that washes out floating (planktonic) microorganisms and coats surfaces with a proteinaceous layer of proteins and glycoproteins; the salivary pellicle. To sustain their growth, oral microbial communities have consequently evolved to behave as “site-specialists”, and to adhere onto tissues (mostly non-shedding dental tissues) at sites that best cover their physiological needs, i.e., their preferred ecological niche [17, 18].

Bacterial adhesion to surfaces, and further intergeneric coaggregations are regulated processes. Adsorbed components of the salivary pellicle, such as statherins, proline-rich proteins, mucins or α-amylase, provide binding motifs that enable the selective attachment of early colonizing species that express cognate surface receptors [19, 20]. These principally include streptococcal species such as *Streptococcus gordonii, Streptococcus sanguinis*, and *Streptococcus mitis* [21]. In turn, early colonizers express at their surface adhesins recognized by *bridging* species, so named as they are themselves exposing surface receptors allowing further incremental coaggregation of later colonizers [22, 23]. *Veillonella* species typically represent such *bridging* taxa and contribute to regulate these intergeneric coaggregations *via* affinity-dependent interactions [24, 25]. The contribution of *bridging* taxa such as *Veillonella* spp. to polymicrobial communities extends beyond the establishment of cell-cell juxtaposition. The metabolism of veillonellae may alter their immediate environment and thereby foster the establishment of more pathogenic species. For instance, though veillonellae are classified as strict anaerobes, their catalase activity was shown to detoxify ambient hydrogen peroxide (H_2_O_2_) stemming from the metabolism of initial colonizers (*Streptococcus*), thereby creating favorable low redox potential conditions for growth by more oxygen-sensitive anaerobes (*Fusobacterium nucleatum*), as would be periodontal pathogens in the gingival sulcus [11]. *Veillonella* was also shown to produce heme, which is the preferred iron source of the periodontopathogen *P. gingivalis* [26]. In these considerations, veillonellae were recently proposed to behave as “accessory pathogens” [23, 27].

## General Characteristics

Veillonellae are strictly anaerobic Gram-negative cocci. The genus *Veillonella* consists of 16 characterized species [28–34], eight of which are routinely isolated from the oral cavity [33, 35, 36]. A unique characteristic of the genus is their lack of glucokinase and fructokinase that renders them unable to metabolize glucose, fructose and disaccharides. Instead, veillonellae use short-chain organic acids as carbon sources, often produced as intermediary/end metabolites from other genera in the biofilm. With the exceptions of *Veillonella seminalis* [28], lactate is the preferred carbon source for most *Veillonella* species, and is metabolized to propionate *via* the methylmalonyl-CoA pathway, and by further pathways to acetate [37]. The metabolic consumption of lactate establishes a nutritional interdependence between *Veillonella* spp. and early-colonizing lactic acid-producing bacteria, such as species of streptococci or lactobacilli, and thereby accounts for their co-localization in oral biofilms [38]. Further worth noting here, is that lactate is the strongest acid produced in significant amount by oral taxa and is involved in the demineralization of dental hard tissues (see section “veillonellae and dental caries” below). Its conversion to weaker volatile acids by *Veillonella* spp. (primarily propionate) may therefore act as a buffer to the carious damages induced by saccharolytic species [20].

Interestingly, despite their strict requirement for anaerobic environments, *Veillonella* spp. demonstrate an important ability to cope with aerobic media. Such observation is partially imputed to their ability to detoxify hydrogen peroxide *via* the expression of a catalase in most *Veillonella* species, with the only exception of *Veillonella atypica*. Altogether, these abilities to establish mutually beneficial networks with both saccharolytic initial colonizers as well as to decrease the redox potential for themselves and later colonizers have made veillonellae among the most abundant taxa in both supra-gingival and sub-gingival biofilms [39–41].

## Taxonomy

**Figure 1:**
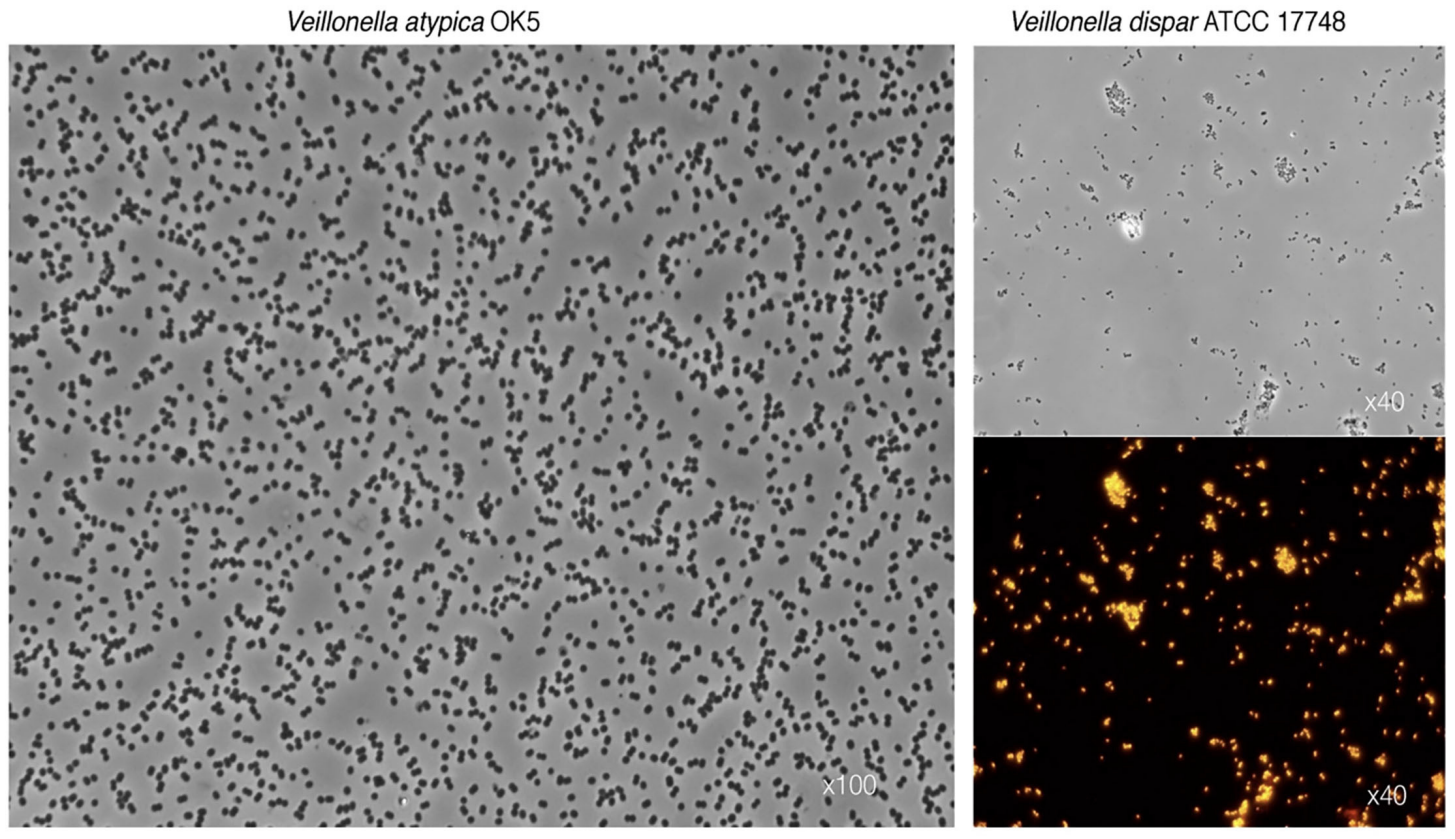
Characteristic photomicrographs of two *Veillonella* species. Differential interference contrast (DIC) observations show the typical diplococcus phenotype for *V. atypica* **(left panel)** and *V. dispar* **(top right panel)**. **Lower right panel** depicts fluorescence *in situ* hybridization (FISH) of the same *V. dispar* cells using the 16S rRNA probe VEI217-Cy3 and epifluorescence microscopy imaging.

Veillonellae belong to phylum *Firmicutes*, and although most members are Gram-positives, representatives of the Class Negativicutes such as *Veillonella* spp. stain Gram-negative. Figure 1 shows typical photomicrographs of two *Veillonella* species as observed under differential interference contrast (DIC) and epifluorescence microscopy using fluorescent probes targeting the 16S rRNA. The genus stems from the Order Veillonellales that also includes the genera *Dialister, Megasphaera*, and *Anaeroglobus*, which have been recently proposed as putative periodontopathogens [42, 43]. Recognized human oral *Veillonella* species include: *Veillonella parvula, Veillonella dispar, V. atypica, Veillonella denticariosi* (reportedly enriched in carious dentine), *Veillonella rogosae* (reportedly enriched in caries-free individuals), *Veillonella tobetsuensis* (often isolated form the tongue), *Veillonella infantium* and *Veillonella nakazawae* [32, 33, 38]. Due to high genomic similarities between species, discrimination among *Veillonella* spp. by the sole use of the 16S ribosomal RNA (16S rRNA) gene, as increasingly applied for taxonomic assignment, may prove highly challenging. Even more so since single genome bears several copies of the 16S gene that may display significant heterogeneities between them (intragenomic variations) [44]. One potential solution may be to complement the 16S-based taxonomy with other housekeeping-gene sequences, such as *rpoB* (encoding for the RNA polymerase β-subunit) and *dnaK* (encoding for Chaperone protein DnaK) [33, 44–46].

## Carbon Source Metabolism in *Veillonella* Species

Lactate and malate are the preferred carbon sources in veillonellae, and are metabolized to propionate, acetate, CO_2_ and H_2_ [37]. Pyruvate, fumarate, and oxaloacetate may also be metabolized, but citrate, iso-citrate and malonate are not. Catabolism of succinate has been reported yet yielding suboptimal growth [47]. The balanced stoichiometry of lactate catabolism is:

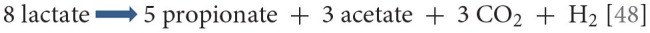



Of great importance in this overall reaction is the unique manner in which energy is produced. The typical generation of an ATP through conversion of pyruvate to acetate occurs, but it is complemented by the action of an enzyme unique to veillonellae: the lactate-oxaloacetate transhydrogenase (or malatelactate transhydrogenase) [49]. This NADH-binding enzyme allows the interconversion of lactate + oxaloacetate to pyruvate + malate in a single reaction without loss of electrons. The ensuing reducing equivalents are used to produce an ATP molecule during the transfer from malate to fumarate. Fumarate is further converted to succinate which is subsequently decarboxylated to generate propionate and carbon dioxide [48]. Thus, this unique transhydrogenase allows more ATP to be generated than would the simple conversion of lactate to pyruvate and to acetate. Another distinctive feature of *Veillonella* metabolism is the regeneration of oxaloacetate from pyruvate by fixation of carbon (from carbon dioxide) *via* the pyruvate carboxylase, rather than through transcarboxylation from methyl-malonyl CoA as occurs in Propionibacteria [50]. The regenerated oxaloacetate can then be decarboxylated and phosphorylated to phosphoenolpyruvate by the phosphoenolpyruvate carboxykinase in order to enter the gluconeogenesis pathway.

An additional source of energy is an atypical pathway of nitrate reduction: nitrate is reduced to nitrite using pyruvate as the electron donor to yield an ATP, and the nitrite is then converted to hydroxylamine, then to ammonia which is assimilated [51]. This unique metabolism endows *Veillonella* spp. with a highly specialized ability to thrive on intermediary/end metabolites produced by other bacterial members in their vicinity.

## Genomics and Genetics

Gronow and his colleagues deposited the first complete genome sequence of a *Veillonella* species, more specifically *V. parvula* strain Te3^T^ (DSM 2008) isolated from a human intestinal tract [52]. The type strain appeared to carry one main chromosome of 2,132,142 bp displaying 38.6% GC content. All predictable 1,920 genes consisted of 1,859 protein coding genes, 61 RNAs and 15 pseudogenes. Among all genes, 73.6% were functionally annotated, and the rest were labeled as hypothetical proteins. More recently, as a result of the mounting interest attracted in oral veillonellae, several other draft and complete genomes were further deposited, these include *V. atypica* OK5, *V. tobetsuensis* ATCC BAA-2400T, *V. parvula* HSIVP1, *V. nakazawae* JCM33966^T^ [36, 53–55].

Despite the purported importance of *Veillonella* in human health and disease, little is known about their biology and pathogenic traits, largely due to our inability to genetically manipulate this group of bacteria until recently. Liu et al. developed a first genetic transformation system in veillonellae, which comprises a genetically transformable strain of *V. atypica* OK5 and a shuttle vector [56, 57]. Using this system, Zhou et al. inactivated by insertion all of the 8 putative hemagglutinin genes in *V. atypica* to study putative surface proteins responsible for coaggregation with oral bacteria [41]. By testing the capacity of all 8 adhesin mutants to coaggregate with various oral bacteria and human oral epithelial cells, one multi-functional adhesin Hag1 was identified in *V. atypica*. This protein is required for *Veillonella* coaggregation with streptococcal species, *P. gingivalis* and human buccal cells, thus believed to play a vital role in the development of human oral biofilm. Based on the same system, Zhou and colleagues successfully established the first markerless mutagenesis system in *V. atypica* using a mutant *pheS* gene (encoding the highly conserved phenylalanyl-tRNA synthetase alpha subunit) as the counter-selectable marker. To study the function of extracellular proteins sigma factor in *Veillonella*, they successfully deleted a gene encoding an alternative sigma factor (*ecf3*) from *Veillonella* chromosomal DNA [58]. This provided a valuable tool for studying genes' function and regulation in *Veillonella* species. Recently, natural competence in *V. parvula* has been reported and this provided a rapid and simple method to study *Veillonella* genetics [45].

## Bridging Role in Oral Biofilm Development

Oral and dental biofilms are multispecies communities, which develop in an incremental process with initial colonizers attaching to surfaces, followed by early- and later-colonizers physically co-aggregating [20, 59]. Evidence that veillonellae function as bridging species in biofilm development stems from both *in vivo* and *in vitro* studies. Human epidemiological studies have shown veillonellae to be highly abundant in both supra- and subgingival plaques as well as on the tongue and in saliva [39, 40, 60–62]. Biofilms formed on intra-oral devices worn by volunteers showed veillonellae colonization as early as 2 h following insertion [63]. The presence of *Veillonella* species so early in biofilm development may appear somewhat surprising considering their requirement for anaerobic conditions and inability to catabolize sugars. Early biofilms are indeed not depauperate in oxygen, and sugars are likely the most abundant carbon source in immature oral biofilms [64]. Although initial colonizing saccharolytic streptococci could supply veillonellae with organic acids from their fermentation metabolism, most of these initially present *Streptococcus* species are not high producers of organic acids. A possible piece in this puzzle rests in the cell-cell recognition and intergeneric interaction [20]. Hughes et al. observed that sub-gingival isolates of the genus have extensive coaggregations with streptococci and *Actinomyces* spp. found in initial plaque [65]. However, isolates from the tongue and other mucosal surfaces display few of these interactions—instead they coaggregate with *Streptococcus salivarius*, an organism that likewise colonizes the tongue, and is less frequently isolated from dental plaque. These results suggest that the phenotype of *Veillonella* isolates may differ based on the isolation site, and that coaggregation interactions may enhance recruitment of a highly adaptable strain to a given microbial community. Coaggregation characteristics and cell-surface antigenic properties (assessed with polyclonal antibodies raised against whole bacterial cells) have been used as phenotypic markers to show that not only do veillonellae occur in immediate proximity to streptococci in the early plaque biofilm, but also that a change in veillonellae phenotypes within a single individual's plaque biofilm occurs over the course of 4 h [66].

Earlier studies have shown that *Veillonella* spp. physically coaggregate with streptococci [65, 67]. Furthermore, *in vitro* studies using saliva as sole medium have demonstrated that when *Veillonella* is co-cultured with later colonizers, such as *F. nucleatum* or *P. gingivalis*, the latter always showed a growth advantage compared to their monocultures [10, 13, 59, 68, 69]. Veillonellae was also found to coaggregate with these two later colonizers, both *in vivo* and *in vitro* [24, 70, 71]. In an *in vitro* multi-species biofilm model, absence of streptococci from the inoculum decreased the total number of *V. dispar* cells [72]. Of note, in the same biofilm model, different *Fusobacterium* spp. differentially affected the growth of *V. dispar*, which may reveal previously unknown nutritional cues between the two taxa [73]. These observations collectively illustrate the bridging role of *Veillonella* spp. within oral biofilms, although the surface proteins and mediators involved in these intergeneric interactions remain largely understudied to date.

Recently, eight putative genes, encoding adhesin proteins, have been identified in *V. atypica* OK5 strain [41]. Among them is a YadA-like autotransporter protein Hag1, encoded by *hag1* gene required for its coaggregation with *S. gordonii, Streptococcus oralis, Streptococcus cristatus, P. gingivalis* and even for adhesion on human oral epithelial cells [41]. Given to the remarkably large size (7,187 amino acids) and complex organization of Hag1 protein, the coaggregation with different partners likely involves distinct domains and mechanisms, which thus far warrant further investigation. Indeed, the target for *V. atypica* OK5 binding to *S. gordonii* has been identified to be Hsa protein, a previously characterized sialic-acid binding adhesin in *S. gordonii* [74].

Phenotypic characterization of bacteria in general has become less prevalent since the advent of modern genomic approaches. However, the aforementioned studies demonstrate that a functional difference between *Veillonella* spp. likely exists, but that we lack an understanding of what the differences may be and how they influence community physiology *in situ*. The question remains as to the carbon source for veillonellae in the early plaque biofilm. Experiments performed in an *in vitro* model show that *V. parvula* PK1910 produces a diffusible signal which upregulates streptococcal amylase production [75]. In turn, degradation of streptococcal intracellular glycogen stores and subsequent glycolysis could yield aberrant lactic acid for the growth advantage of veillonellae. This observation is all the more important because the model system has incorporated flow. Neither signaling nor fermentation products can be permitted to accumulate, therefore these components can be present at a consistently high concentration only within the nearest proximity to the producer bacterial cell. Accordingly, only those streptococci in immediate proximity to veillonellae may be “signal-activated,” and, reciprocally, only those veillonellae in immediate proximity to streptococci would benefit from any enhanced lactate production. While these interactions are limited to dual bacterial species assays, recent advents in omics hold great promise for determining how *Veillonella* establishes mutualistic relationships within bacterial communities in niches that are characterized by *Veillonella* overgrowth. Thus, a convergence of coaggregation, physiology, and solute concentration gradients may influence which veillonellae are found at the various sites in healthy and diseased individuals.

## Veillonellae and Dental Caries

Dental caries, i.e., the demineralization of teeth enamel and dentine, are mainly caused by the local decrease of pH induced by the production of organic acids ensuing from fermentation in saccharolytic bacteria [5]. The mouth encompasses approximately 700 microbial species, but only a few specific species have been consistently implicated in dental caries, such as *Lactobacillus* spp. and *S. mutans* [76]. Yet, more recent studies using next generation sequencing identified additional taxa, most consistently *Scardovia wiggsiae*, and mixed taxonomic communities with common saccharolytic functions in the etiology of the disease [77–79].

Early studies by Mikx and colleagues on the role of oral bacteria in caries activity showed that *Veillonella alcalescens* may display a potentially anti-cariogenic effect [80]. Indeed, gnotobiotic rodents infected with *S. mutans* and/or *V. alcalescens* demonstrated less dental caries development when co-inoculated with both taxa. One later report came to challenge the purported role of veillonellae in dental health [81]. Using removable dental appliances worn by caries-free and caries-susceptible volunteers, Minah et al. showed that frequent exposure to sucrose stimulated the growth of *Veillonella* spp., *Lactobacillus* spp., *S. salivarius*, and to a lesser extent, *S. mutans*, and further decreased the microhardness of enamel. Whereas, this positive association appears to associate *Veillonella* spp. with caries development, it may also be reasoned that *Veillonella* spp. were advantageously thriving on the increased lactic acid production generated by streptococci and lactobacilli following sucrose exposure. Moreover, the authors argued that the increased level of *Veillonella* in caries-free patients' plaque exposed to sucrose, is consistent with its protective role in enamel decalcification. By contrast, Noorda et al. established an artificial mouth model using human enamel slabs and found co-cultures of *S. mutans* and *V. alcalescens* to generate an increased acid production as compared to the respective monocultures. Co-cultures also resulted in higher enamel surface demineralization under anaerobic conditions [82]. Beyond the consistent observation that *Veillonella* spp. are associated with dental caries, it appears difficult to conclude whether such association is causal or consequential in consideration of these conflicting findings.

Not only in childhood group, were *Veillonella* spp. (especially *V. parvula*) found to be associated with caries [83], but in elder population, they are also one of the most abundant and prevalent in all samples from both health and caries, furthermore, the abundance from caries appeared to be higher [84]. Evidence exists that *V. denticariosi* occurs only in diseased sites whereas, *V. rogosae* is found only in healthy plaque [85], i.e., that *V. denticariosi* has unique properties which either restrict it to a caries community or make it uncompetitive elsewhere. One may ask whether *V. denticariosi* isolates from caries lesions display interactions with mutans streptococci? The physiological link between veillonellae (as lactate utilizers) and mutans streptococci (as lactate producers) has prompted much clinical research on the association of veillonellae with caries. For instance, Backer et al. [40] utilized a reverse capture checkerboard assay to analyze plaque samples from 30 subjects with caries together with 30 healthy controls and found *Veillonella*, together with 7 other species including *S. mutans*, to be associated with caries. A study by Aas et al. [86] also demonstrated the association of genera *Veillonella* with caries progression. Belstrom et al. reported that *Streptococcus* spp. and *Veillonella* spp. were the most predominant genera among all saliva samples from 292 participants with mild to moderate dental caries [87].

It may be purported that the association observed between cariogenic bacteria and veillonellae stems from their metabolic requirement for organic acids that are indeed found in higher concentrations in active caries. Hence, the presence of veillonellae may be indicative, and perhaps predictive, of a localized drop in pH. Indeed, by using an chemostat and a 9-species microcosm biofilm, Bradshaw and Marsh reported that numbers and proportions of *S. mutans* and *Lactobacillus* spp. increased as pH decreases, while *V. dispar* became the most abundant organism following glucose pulses, especially under low pH [88]. Similarly in another clinical study relying on community 16S cloning and sequencing, Gross et al. found the proportion of veillonellae to increase commensurately with the proportion of acidogenic streptococci [89]. At the very least, these studies suggest that veillonellae are spatiotemporally associated with acidogenic bacteria in oral biofilms, and it is enticing to consider that their correlation with caries progression identifies this genus as an early indicator of dysbiotic communities, regardless of the accompanying acidogenic phylotypes. This does not necessarily implicate veillonellae as etiological agents of dental caries, but ecological beneficiaries of an incipient dysbiotic community. In other words, veillonellae could comprise a risk factor for caries initiation, while mutans streptococci a risk factor for caries progression.

## Veillonellae and Periodontitis

Periodontitis affects about 11% of the population around the world and is a biofilm-induced chronic inflammatory disease that causes resorption of the periodontium, i.e., teeth-supporting tissues [90, 91]. According to current and most accepted knowledge on periodontitis etiopathogenesis, the initial phase of the disease (gingivitis) is triggered by the accumulation of supra- and sub-gingival biofilm [92]. Rapidly the accumulation of bacterial cells and metabolites induces a mild inflammation that in turn results in an increased exudation of gingival crevicular fluid [92]. As a serum transudate/exudate, excess gingival fluid creates a protein-rich environment that fosters colonization by proteolytic species. One common view to explain the subsequent progression to periodontitis relies on the keystone-pathogen hypothesis, that purports low-abundant taxa of the red complex pathogens (*P. gingivalis, T. forsythia*, and *T. denticola*) as well as *F. nucleatum*, to further alter local nutrient conditions by tissue breakdown, subvert host immunity and ultimately promote the establishment of more abundant pathogens [93–95]. These microbial alterations set-off a self-feeding vicious cycle that enhances and maintains periodontal inflammation and leads to tissue resorption.

All periodontal pathogens are considered as “intermediate” (*F. nucleatum*) or later (*P. gingivalis*) colonizing bacteria, and regularly colonize the subgingival crevice, which is mostly an anaerobic environment. This is consistent with the fact that these periodontal pathogens are obligate anaerobes and therefore extremely vulnerable to oxydative stress. Reactive oxygen species (ROS), such as hydrogen peroxide (H_2_O_2_), are commonly generated by the metabolism of initial colonizers and may inhibit the growth of strict anaerobes, such as periodontopathogens [59, 96–99]. An interesting and contradictory observation showed that *F. nucleatum* and *P. gingivalis* are frequently isolated even from early biofilm communities [100]. How do these strictly anaerobic periodontopathogens cope with this lethal H_2_O_2_ concentrations likely to occur around streptococci? As early colonizer, *V. parvula* has been reported to be catalase-positive bacterium and able to eliminate H_2_O_2_ in the microniche and then rescue the growth of these anaerobic periodontopathogens [11]. Thus, the facts that *Veillonella* are frequently identified in the healthy oral microbiome and not considered as periodontal pathogen cannot rule out the possibility that this species greatly contributes to the shift from health to gingivitis and finally to periodontitis.

**Figure 2:**
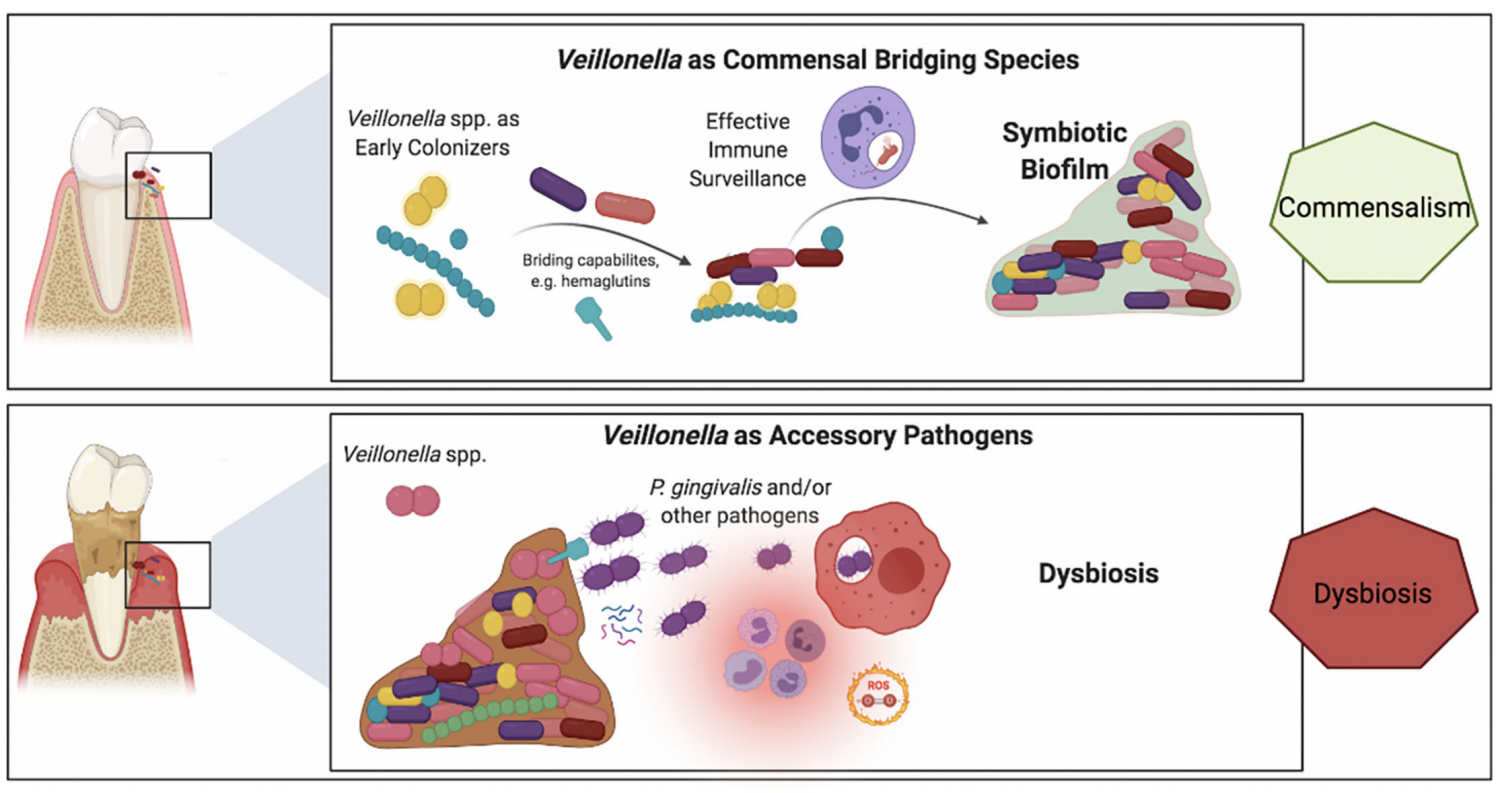
Schematic representation of the purported roles of *Veillonella* spp. in a commensal biofilm associated with health vs. a dysbiotic microbial community associated with periodontitis. *Veillonella* spp. are highly abundant within oral biofilms and besides their well-established bridging role in commensal biofilms (top panel), their alternative ecological role of as accessory pathogens has recently emerged (lower panel). **Upper panel:** *Veillonella* spp. have a key role in the colonization of mineralized (e.g., tooth enamel or cementum) and metal (e.g., dental implants) surfaces within the oral environment. Demonstrating symbiotic mutualism with commensal streptococci, they are early and abundant colonizers that also provide immune stimulation by their mildly potent LPS, which may be beneficial to the host for heightened immune surveillance that contributes to the establishment of a dynamic healthy equilibrium. **Lower panel:** In the inflammatory environment of the crevice in the presence of biofilms with high plaque biomass the microenvironmental conditions vary within the biofilm. Differences in lactate concentrations, the prime carbon source for *Veillonella*, and differences in oxygen availability may trigger virulent mechanisms in certain *Veillonella* strains, such as hemagglutinin-1 that provide adhesion positions for *P. gingivalis* or small molecules that support *P. gingivalis* early growth and colonization.

Whereas, compelling evidence supports the keystone pathogen hypothesis [101], the fact that low-abundant *P. gingivalis* communities exhibit difficulties to sustain themselves and to durably colonize the oral cavity may indicate interdependence with a yet-missing piece of the puzzle [102, 103]. It is well-known that *P. gingivalis* requires hemin or heme for growth, and this nutrient is provided by the crevicular fluid during gum inflammation [104, 105]. *P. gingivalis* can be found in the early dental biofilm, where no inflammation occurs [100]. Recently, Zhou et al. reported that *V. atypica*, as early colonizer, can generate hemin/heme to support *P. gingivalis* growth *in vitro* [26]. This study suggests that *Veillonella* could be a potential hemin/heme provider to support periodontopathogen growth and might play a crucial role in dental biofilm formation and periodontitis development. In addition, unlike early-colonizers, *P. gingivalis* is indeed unable to grow at low-cell densities and does require large initial inocula [106]. Most recently, it has been shown that the presence of *V. parvula* in co-cultures also supports the growth of *P. gingivalis*, even when the latter was inoculated at low-cell densities [23]. The mechanism of directly physical interaction between *Veillonella* and *P. gingivalis* has been reported by Zhou et al. [41], but this growth promoting signal appeared not to be dependent on cell-cell contact and rather was mediated *via* a soluble factor. More interestingly, this soluble factor was necessary to enable the colonization by *P. gingivalis* of a mice model oral cavity [23]. Although it remains unclear whether this growth-promoting factor acts as a quorum-sensing signal informing on cell density, a nutrient necessary to *P. gingivalis* or simply a metabolic mediator, these findings highlight the *bridging* role of *Veillonella* spp. in biofilm microbial interactions and illustrate the concept of “accessory pathogen”. Figure 2 provides a schematic representation of this dual role of *Veillonella* spp. within oral biofilms, at times commensals yet also potentially pathogenic.

## Future Perspective

Considering the important abundance of the genus *Veillonella* in the oral cavity, the limited number of studies available until recently is surprising. Potential reasons that may account for this scarcity include the commensal nature of *Veillonella* spp., together with the lack of dedicated genetic tools that lately allowed deeper investigation of the genus. More recently, an increasing number of studies have pointed toward the importance of veillonellae in the ecology of oral biofilms and their role in the homeostasis between oral health and disease.

First, there is now compelling evidence showing that *Veillonella* spp., as bridging organisms, play a pivotal role in establishing multispecies biofilm communities *via* direct and indirect interactions with both initial and later colonizers [9, 10, 13, 41, 74]. The recent development of genetic tools in *Veillonella* spp. [45, 56–58], the mechanisms of *Veillonella* binding with other oral bacteria and human epithelial cells have been studied [41, 74]. However, due to the complexity of veillonellae's outer membrane and abundance of adhesins, further studies are warranted to better understand veillonellae's role in the development of multispecies biofilm communities.

Second, it is crucial that future research focuses on veillonellae's role as “accessory pathogen” in incipient dysbiosis. Indeed, while their pathogenic potential has been shown to be limited, the role of *V. atypica* in producing nutrients and reducing the oxidative microenvironment to support and facilitate the growth of periodontal pathogens has been reported [11, 26]. Most recently, Hoare and his colleagues reported that *P. gingivalis*, even inoculated at low-cell densities, also can survive in co-culture with *V. parvula* [23]. In addition, *Veillonella* spp. are spatiotemporally associated with acidogenic bacteria in oral biofilms and caries development, and then identified as an early indicator of dysbiotic communities in dental caries [89]. Considering the fact that veillonellae are early colonizer and frequently isolated and identified in early biofilm communities, the studies investigating their role in the development of oral diseases, such as periodontitis and caries, and characterize their putative involvement as “accessory pathogen” may have significant clinical relevance.

Third, it is important to study *Veillonella*'s biology at a species- and strains-level resolution, rather than at the genus level. Most studies that investigated the ecological role of veillonellae in oral biofilms often remained species-specific. As an example, it has been reported that *V. denticariosi* is only identified in caries sites whereas, *V. rogosae* is isolated in healthy plaque [85]. Another instance, the spectrum of *Veillonella* interspecies interaction with oral microbes showed that among all tested *Veillonella* strains, all *V. parvula*, partial *V. atypica* and *V. rogosae*, and none of *V. dispar* physically interact with *S. gordonii* [74]. Whereas, these studies emphasize differences observed at the species level, different strains from the same species often display further genomic variations [107]. As such, various *Veillonella* strains may display different abilities to behave as commensals or conversely as “accessory pathogens” that remain concealed at the strain-level. However, there is a big challenge for *Veillonella* research in the future. So far, the genetic system has only been established in *V. atypica* and *V. parvula*, this is because the most species in the *Veillonella* genus are non-transformable [45, 57, 58]. Isolating and establishing genetic tools in different *Veillonella* species/strains will be crucial in the coming years.

Although *Veillonella* are frequently identified in the healthy oral microbiome and not considered as periodontal pathogen, is it possible that they are pathobionts for other oral diseases? Most recently, Daubert et al. reported that peri-implantitis is associated with a significant increase in *Veillonella* spp. [108]. Peri-implantitis is an infectious disease that causes an inflammatory process in soft and hard gum tissues around dental implants [109, 110]. In peri-implantitis, titanium corrosion and dissolution has been implicated and titanium particles are generated in disease progression [111, 112]. The enrichment of *Veillonella* genus in the diseased peri-implant microbiome has been found to be correlated to the local concentration of titanium particles in the crevice, which titanium particles modify the peri-implant microbiota toward dysbiosis [108]. Because titanium particles in peri-implantitis strongly activate oxidative burst pathways in humans [113], the potential role of *Veillonella* spp. with capabilities to detoxify reactive oxygen species (e.g., through catalase activity) may have important roles in microbiome dysbiosis in titanium-mediated inflammation in peri-implantitis. This connection warrants further investigation to better understand the differences between the periodontal and peri-implant microbiome.

In addition, it has been reported that periodontal disease is associated with atherosclerosis, and this might be because oral bacteria contribute to the progression of atherosclerosis and cardiovascular disease [114]. Koren et al. reported that the genera *Veillonella* and *Streptococcus* are identified in the majority atherosclerotic plaque samples, and the combined abundances of these two taxa in atherosclerotic plaques are consistent with their abundance in the oral cavity, implying that *Veillonella* spp. might play a crucial (possibly potential pathogenic) role in the development of atherosclerosis and cardiovascular disease [115]. Thus, as a potential pathobiont, *Veillonella*'s role in the development of other diseases remains to be investigated in future.

## Author Contributions

PZ and DM drafted the manuscript. GB and GK provided critical feedback. All authors contributed to figure development and have reviewed and approved the revised manuscript.

## Conflict of Interest

The authors declare that the research was conducted in the absence of any commercial or financial relationships that could be construed as a potential conflict of interest.

## Publisher's Note

All claims expressed in this article are solely those of the authors and do not necessarily represent those of their affiliated organizations, or those of the publisher, the editors and the reviewers. Any product that may be evaluated in this article, or claim that may be made by its manufacturer, is not guaranteed or endorsed by the publisher.
